# Genetic Analysis Workshop 17 mini-exome simulation

**DOI:** 10.1186/1753-6561-5-S9-S2

**Published:** 2011-11-29

**Authors:** Laura Almasy, Thomas D Dyer, Juan Manuel Peralta, Jack W Kent, Jac C Charlesworth, Joanne E Curran, John Blangero

**Affiliations:** 1 Department of Genetics, Texas Biomedical Research Institute, 7620 NW Loop 410, San Antonio, TX 78245, USA; 2 Centro de Investigación en Biología Molecular y Celular, Universidad de Costa Rica, San José, Costa Rica; 3 Menzies Research Institute, 17 Liverpool St (Private Bag 23), Hobart, Tasmania 7001, Australia

## Abstract

The data set simulated for Genetic Analysis Workshop 17 was designed to mimic a subset of data that might be produced in a full exome screen for a complex disorder and related risk factors in order to permit workshop participants to investigate issues of study design and statistical genetic analysis. Real sequence data from the 1000 Genomes Project formed the basis for simulating a common disease trait with a prevalence of 30% and three related quantitative risk factors in a sample of 697 unrelated individuals and a second sample of 697 individuals in large, extended pedigrees. Called genotypes for 24,487 autosomal markers assigned to 3,205 genes and simulated affection status, quantitative traits, age, sex, pedigree relationships, and cigarette smoking were provided to workshop participants. The simulating model included both common and rare variants with minor allele frequencies ranging from 0.07% to 25.8% and a wide range of effect sizes for these variants. Genotype-smoking interaction effects were included for variants in one gene. Functional variants were concentrated in genes selected from specific biological pathways and were selected on the basis of the predicted deleteriousness of the coding change. For each sample, unrelated individuals and family, 200 replicates of the phenotypes were simulated.

## Background

The state of the science for localization and identification of genes that influence common complex diseases has changed rapidly over the past 20 years. As laboratory costs continue to fall with the development of more efficient high-throughput techniques, the field is quickly proceeding toward studies that make use of genome-wide sequence data. There is as yet no consensus on optimal, or even appropriate, statistical genetic approaches for analyzing exome sequence data, and few investigators have had experience analyzing such data sets. This was the motivation for the Genetic Analysis Workshop 17 (GAW17) “mini-exome” data set. The GAW17 data set is a hybrid of simulated and real data. Real exome sequence data from the 1000 Genomes Project were used as the basis for simulating a common complex disease and related quantitative risk factors. Two different study designs were simulated, unrelated individuals and large families, each with the same sample size.

### 1000 Genomes Project

The 1000 Genomes Project (http://www.1000genomes.org) is designed to survey genetic variation at the sequence level across multiple human population groups. It includes individuals of European, East Asian, South Asian, West African, and American Indian ancestry. Three pilot projects for the 1000 Genomes Project were completed in 2010: low-fold genome-wide sequencing of 179 individuals, higher fold sequencing of two parent-child trios, and exonic sequencing in 697 individuals [1]. Publicly available exon sequence data from the 1000 Genomes Project were used to provide a realistic pattern of number and frequency of single-nucleotide polymorphisms (SNPs), including cross-population variation and linkage disequilibrium between sites, for the GAW17 simulations.

## Methods

### Genotype calling

SNP genotypes were obtained from the sequence alignment files provided by the 1000 Genomes Project for their pilot3 study. When the GAW17 data set was generated, the 1000 Genomes Project had not yet posted processed calls of these genotypes for each individual. Thus the UnifiedGenotyper method from the Genome Analysis Toolkit (GATK) package [2] was used for the detection of SNPs and for the calling of SNP genotypes. A male human genome based on National Center for Biotechnology Information reference sequence 36 (RefSeq36) human genome release (human_b36_male.fasta.gz) was used as the reference genome sequence for both male and female alignments.

The UnifiedGenotyper method was run twice on the alignment files. The first time it was allowed to scan freely through the alignments to search for variation against the reference sequence to be considered as SNP candidates. Genotypes that were not homozygous for the reference base allele were called for the candidate SNPs detected. Because of time and technical constraints, GAW17 SNPs were chosen to be the subset of candidate SNP genotypes that were called from an alignment of 10 or more sequencing reads. During the second run, genotypes, including those homozygous for the reference base, were called only for the subset of SNPs selected in the first run.

This procedure had the advantages of being fast, correctly calling most of the true common SNP variants, generating a large volume of rare SNP variants, and producing a genotype matrix with few missing genotypes to simplify downstream preparation of the simulated data set. However, it was not meant to detect the true natural variation present in the 1000 Genomes Project. Thus there were more rare SNPs in the GAW17 data set than those described in the 1000 Genomes Project Consortium analyses of their own pilot data sets [1]. The enrichment of rare variants in the GAW17 data set was caused in part by artifacts introduced by, for example, lack of filtering.

The 1000 Genomes Project genotypes were not phased, and some genotypes were missing as a result of incomplete sequence coverage in some individuals. We used the program fastPHASE [http://depts.washington.edu/uwc4c/express-licenses/assets/fastphase/] to infer missing genotypes and haplotypic phase. In the family data set (described later), we used the program CHRSIM [3] to drop the phased founder haplotypes throughout the rest of the pedigree. Recombination was taken into account, with a single obligate crossover event occurring on each chromosome.

As noted, the GAW17 genotypes differ from the official 1000 Genomes Project called genotypes for the same individuals because of differing approaches to genotype calling, inclusion or exclusion of regions of low-fold coverage, and the inclusion of the imputed genotypes in the GAW17 data set. Imputed genotypes were not identified as such in the distributed data and were treated as equally “real” as called genotypes in the phenotype simulations. These choices were motivated by a focus on designing a data set that would be useful for developing methods related to gene localization, identification, and characterization, with the 1000 Genomes Project data primarily serving as a source of sequence data with realistic patterns of SNP distribution, allele frequency, population variation, and linkage disequilibrium. However, these decisions limited the utility of the GAW17 data for population genetic analyses or for examination of effects of genotype calling or data cleaning on gene finding.

### Distributed genotype data

**Figure 1:**
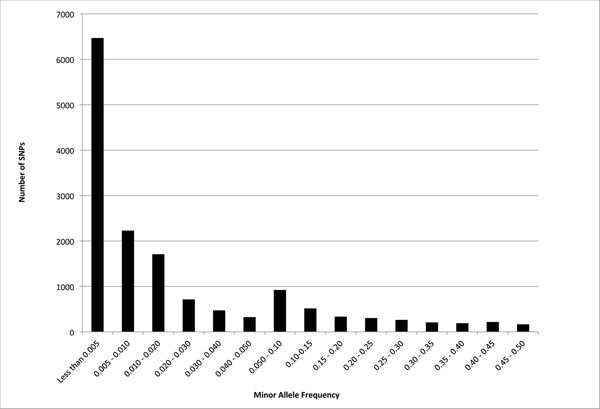
**Minor allele frequency in the unrelated individuals sample for the 15,054 SNPs present in multiple copies**. Note that the scale of the MAF categories is uneven, going by 0.5% intervals for MAF < 0.01, by 1% intervals for MAF = 0.01–0.05, and by 5% intervals thereafter.

The called genotype data distributed for GAW17 included the inferred genotypes, such that all individuals had genotypes for all base-pair positions, and phenotypes were simulated on the basis of these data. Markers were numbered sequentially on each chromosome and were labeled C#S# (e.g., C1S254 is the 254th SNP on chromosome 1); marker locations were recorded as RefSeq36 base-pair coordinates. The 24,487 autosomal SNPs detected in genotype calling were, for purposes of the simulation, assigned to 3,205 genes based on the first intersection found of the marker location and the base-pair coordinates of all genes obtained from RefSeq36 annotations. SNPs that overlapped multiple genes were assigned to only one of those genes. There were 1 to 231 SNPs per gene (mean = 7.64, SD = 14.00). Of the SNPs, 9,433 (38.4%) were private variants, occurring once in the set of 697 unrelated individuals. Multiple private variants carried by the same individual resulted in SNPs with identical genotypes, including a SNP in the *KDR* gene that was designated as functional in the phenotype simulations which had identical genotypes to multiple nonfunctional SNPs. Relatively few of the variants were common; 74% had minor allele frequency (MAF) ≤ 0.01 and only 12.8% had MAF ≥ 0.05 (Figure 1). The median MAF was 0.002, that is, three copies of the minor allele in the sample of 697 unrelated individuals.

### Unrelated individuals and pedigree samples

Two disparate sampling designs were used in the construction of the simulated data. One sample consisted of 697 unrelated individuals, each of whom corresponded to an individual from the 1000 Genomes Project data. The 1000 Genomes Project subjects whose data were used came from the CEPH (European-descent residents of Utah; *n* = 90), Denver Chinese (*n* = 107), Han Chinese (*n* = 109), Japanese (*n* = 72), Luhya (*n* = 108), Tuscan (*n* = 66), and Yoruban (*n* = 112) population groups. The second sample configuration used in GAW17 simulations consisted of 697 individuals in 8 extended families with the genotypes for the 202 pedigree founders being taken from the 1000 Genomes Project data. The founders of the pedigrees were chosen at random from the unrelated individuals sample and included 12 CEPH, 18 Denver Chinese, 19 Han Chinese, 28 Japanese, 50 Luhya, 66 Tuscan, and 9 Yoruban samples. Because of a computational error, the genotypes of pedigree founders were merged incorrectly across files, resulting in an incomplete match between the genotypes of the pedigree founders and the corresponding individual from the unrelated individuals sample. This affected a small proportion of genotypes (7%) but impacted all pedigree founders. Approximately one-third of the SNPs were unaffected, and for the two-thirds that had substitutions, most had only one or two founders with altered genotypes. Pedigree configurations were adapted from the pedigrees used for simulated data in Genetic Analysis Workshops 10 and 12 [4,5] and included four generations and relatives as distant as second cousins. The data set was designed such that all family members had genotype and phenotype data available with no missing or unexamined relatives.

Because the pedigree founders were a subset of the unrelated individuals, genetic diversity was restricted in the families compared to the unrelated individuals sample. Of the 24,487 variant sites identified in the unrelated individuals sample, 10,703 were monomorphic in the family sample with only one allele appearing. On the other hand, some variants that were present in single copies or at low frequency in the unrelated individuals sample appeared many times in the family sample, because they were transmitted by a founder with numerous descendants. For example, C6S2981, which was designated as functional in the phenotype simulations, was present in 3 copies in the unrelated individuals sample and in 46 copies in the family sample. C4S4935, also designated as functional in the simulations, was present in a single copy in the unrelated individuals sample but in 31 copies in the pedigree sample.

There are 327 males and 370 females in the unrelated individuals data set, which preserved the listed sex for each of the 1000 Genomes Project samples. The family set included 346 males and 351 females. Pedigree founders were allowed to have a different sex from the unrelated individuals whose genotypes they shared. However, only autosomal markers were used in the GAW17 simulations (i.e., X and Y data were not included). Assigned ages were matched across the family and unrelated individuals data sets and ranged from 16 to 91 years, with a mean of 41.8 years.

For the family data set, fully informative markers were generated at each gene (recombination was not allowed within genes) and used to compute identical-by-descent (IBD) allele sharing at each gene location under the rationale that family-based data sets were likely to have previous short tandem repeat (STR) or high-density SNP genotyping that could be used to estimate the IBD allele sharing. These IBD matrices were provided as part of the GAW17 data set.

### Phenotype model

A common disease, with a prevalence of 30%, was simulated along with three related quantitative risk factors, Q1, Q2, and Q4. Smoking status (prevalence 25%) was also simulated. Phenotype simulations were performed multiple times to generate 200 replicates of the unrelated individuals and pedigree data sets. Note that the genotype data remained constant across replicates, as did age, sex, and pedigree configuration.

Knowledge about biological pathways and statistical predictions regarding the potential deleteriousness of coding variants was used in designing the simulation model. Pathways for gene enrichment were selected from the publicly available Kyoto Encyclopedia of Genes and Genomes (KEGG) database (http://www.genome.jp/kegg/) and the proprietary software Ingenuity Pathways Analysis (IPA), version 8.7 (http://www.ingenuity.com). The vascular endothelial growth factor (VEGF) pathway was observed to have numerous genes with available typed SNPs and was therefore selected as the source of a subset of the functional loci for phenotype simulations. The IPA version of the VEGF signaling pathway was used as the core source because it included most of the genes in the KEGG VEGF signaling pathway as well as some additional upstream information, primarily relating to VEGF transcriptional control. The overlap between the two data sources was considered significant enough not to impede the investigations of any researchers limited to the freely available KEGG data set.

**Figure 2:**
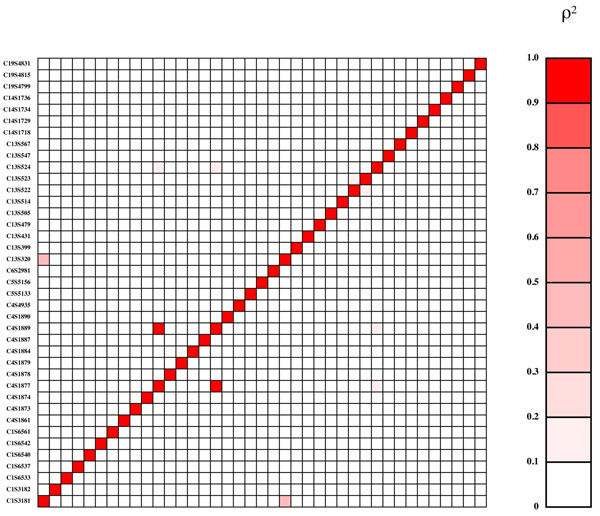
**Gametic disequilibrium (*r*^2^) between functional variants for Q1**. Markers are shown in chromosomal order from bottom to top and from left to right and are symmetric across the diagonal.

**Figure 3:**
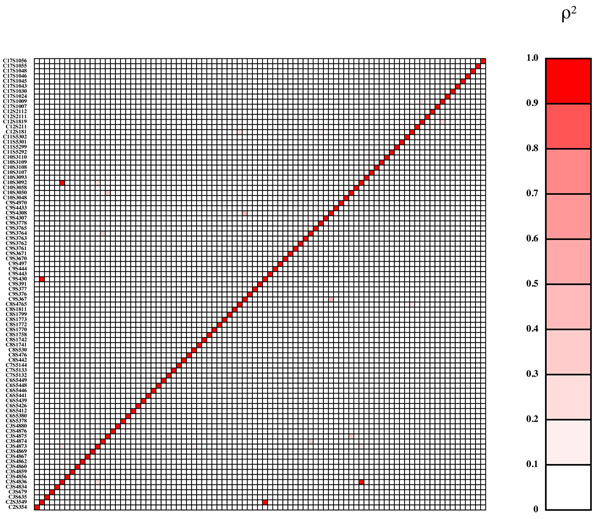
**Gametic disequilibrium (*r*^2^) between functional variants for Q2**. Markers are shown in chromosomal order from bottom to top and from left to right and are symmetric across the diagonal.

**Figure 4:**
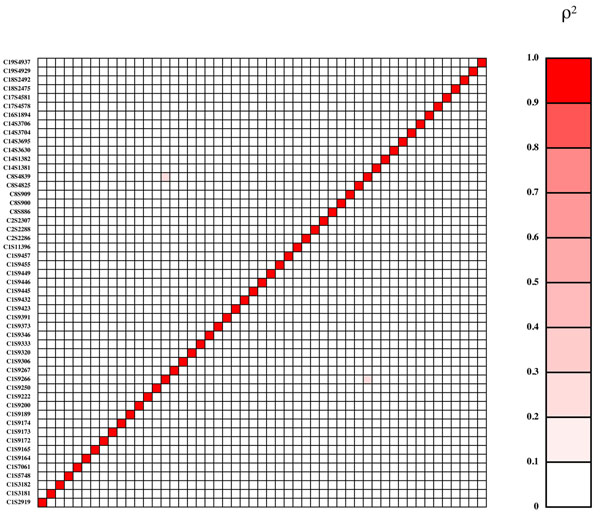
**Gametic disequilibrium (*r*^2^) between functional variants for latent liability**. Markers are shown in chromosomal order from bottom to top and from left to right and are symmetric across the diagonal.

Genes influencing Q1 come primarily from the VEGF pathway; those influencing Q2 were chosen without reference to pathways and were primarily related to cardiovascular disease risk and inflammation, and those influencing latent disease liability also came primarily from the VEGF pathway (a different section from the one in which genes were selected for Q1). Effect sizes for coding variants within genes were assigned using PolyPhen and SIFT predictions of the likelihood that the variant would be deleterious. The functional variants included both rare and common alleles and a range of effect sizes, with most having small effects but a few having large effects that should be reliably detectable in most replicates of the data set. Some genes contained a single functional variant and others contained many. Population origin of the 1000 Genomes Project participants was not used in the phenotype simulations. In general, there was little disequilibrium between the functional variants (Figures 2, 3, 4), with a few exceptions that were primarily private variants carried in a single copy by the same individual (e.g., C3S4836 and C10S3092 for Q2 and C4S1877 and C4S1889 for Q1).

Quantitative risk factors Q1, Q2, and Q4 were simulated as normally distributed phenotypes. Disease was simulated using a liability threshold model, and the top 30% of the distribution was declared affected. All SNP effects were additive on the quantitative trait or liability scale, with each copy of the minor allele increasing the mean trait value by an equal amount. Genotype by environment effects were simulated for Q1. Because genotype, age, and sex were held constant across replicates, the variation in phenotype across replicates came primarily from the residual polygenic and residual environmental components. The residual polygenic components were correlated between relatives, by definition, and also correlated between Q1, Q2, and latent liability. The residual environmental components were unique to each individual and were simulated to be weakly correlated between Q1, Q2, and latent liability.

### Q1

Quantitative risk factor Q1 was influenced by 39 SNPs in 9 genes (see Table 1). There were 1–11 functional variants per gene. Their MAFs in the 1000 Genomes Project data ranged from 0.07% (i.e., a single copy of the minor allele) to 16.5%. In all cases, the minor allele was associated with higher mean Q1; the *β* column in the table provides the displacement in mean levels of Q1 for each copy of the minor allele. Q1 also had a residual heritability of 0.44, resulting from variants at loci not included in the current sequence data set. The residual genetic component of Q1 was correlated with the residual genetic components of Q2 and latent liability. There were also weaker environmental correlations between Q1 and Q2 and latent liability. Values of Q1 were higher in smokers, and there was genotype by smoking interaction for the effects of variants in the *KDR* gene on Q1. Effects of the *KDR* variants were 50% higher in smokers than in nonsmokers. (Note that for *KDR* the effect sizes given in Table 1 are those for nonsmokers.) Q1 also increased with age.

**Table 1:** Effects on Q1

Gene	SNP	MAF	*β*
*ARNT*	C1S6533	0.011478	0.589734
*ARNT*	C1S6537	0.000717	0.642689
*ARNT*	C1S6540	0.001435	0.323662
*ARNT*	C1S6542	0.002152	0.488219
*ARNT*	C1S6561	0.000717	0.625721
*ELAVL4*	C1S3181	0.000717	0.795093
*ELAVL4*	C1S3182	0.000717	0.328748
*FLT1*	C13S320	0.001435	0.18047
*FLT1*	C13S399	0.000717	0.457361
*FLT1*	C13S431	0.017217	0.732566
*FLT1*	C13S479	0.000717	0.839669
*FLT1*	C13S505	0.000717	0.38582
*FLT1*	C13S514	0.000717	0.549816
*FLT1*	C13S522	0.027977	0.623466
*FLT1*	C13S523	0.066714	0.653351
*FLT1*	C13S524	0.004304	0.596704
*FLT1*	C13S547	0.000717	0.549214
*FLT1*	C13S567	0.000717	0.0905862
*FLT4*	C5S5133	0.001435	0.120761
*FLT4*	C5S5156	0.000717	0.385374
*HIF1A*	C14S1718	0.000717	0.251622
*HIF1A*	C14S1729	0.002152	0.329088
*HIF1A*	C14S1734	0.012195	0.220448
*HIF1A*	C14S1736	0.000717	0.228202
*HIF3A*	C19S4799	0.000717	0.174668
*HIF3A*	C19S4815	0.000717	0.51468
*HIF3A*	C19S4831	0.000717	0.265181
*KDR*	C4S1861	0.002152	0.598271
*KDR*	C4S1873	0.000717	0.715613
*KDR*	C4S1874	0.000717	0.503025
*KDR*	C4S1877	0.000717	1.17194
*KDR*	C4S1878	0.164993	0.149975
*KDR*	C4S1879	0.000717	0.610938
*KDR*	C4S1884	0.020803	0.318125
*KDR*	C4S1887	0.000717	0.312058
*KDR*	C4S1889	0.000717	1.17194
*KDR*	C4S1890	0.002152	0.417977
*VEGFA*	C6S2981	0.002152	1.13045
*VEGFC*	C4S4935	0.000717	1.40529

### Q2

Q2 was influenced by 72 SNPs in 13 genes (see Table 2). There were 1–13 functional variants per gene. MAFs ranged from 0.07% to 17.07%. In all cases, the minor allele was associated with higher mean Q2. Q2 had a residual heritability of 0.29. The residual genetic component of Q2 was correlated with the residual genetic components of Q1 and latent liability. There were also weaker environmental correlations between Q2 and Q1 and latent liability. Q2 was not influenced by age, sex, or smoking status.

**Table 2:** Effects on Q2

Gene	SNP	MAF	*β*
*BCHE*	C3S4834	0.000717	0.232562
*BCHE*	C3S4836	0.000717	0.352589
*BCHE*	C3S4856	0.000717	0.311344
*BCHE*	C3S4859	0.002152	0.557489
*BCHE*	C3S4860	0.000717	0.339017
*BCHE*	C3S4862	0.000717	0.93321
*BCHE*	C3S4867	0.000717	0.67704
*BCHE*	C3S4869	0.000717	1.15994
*BCHE*	C3S4873	0.002869	0.588113
*BCHE*	C3S4874	0.000717	1.06857
*BCHE*	C3S4875	0.000717	1.15207
*BCHE*	C3S4876	0.000717	0.798247
*BCHE*	C3S4880	0.001435	0.164995
*GCKR*	C2S354	0.012195	0.396642
*INSIG1*	C7S5132	0.000717	0.0983783
*INSIG1*	C7S5133	0.000717	0.106056
*INSIG1*	C7S5144	0.000717	0.237783
*LPL*	C8S442	0.015782	0.490165
*LPL*	C8S476	0.000717	0.725673
*LPL*	C8S530	0.001435	0.800024
*PDGFD*	C11S5292	0.008608	0.60155
*PDGFD*	C11S5299	0.000717	0.823159
*PDGFD*	C11S5301	0.000717	0.982146
*PDGFD*	C11S5302	0.001435	0.814925
*PLAT*	C8S1741	0.003587	0.71858
*PLAT*	C8S1742	0.000717	0.891241
*PLAT*	C8S1758	0.001435	0.86814
*PLAT*	C8S1770	0.000717	0.58405
*PLAT*	C8S1772	0.001435	0.219187
*PLAT*	C8S1773	0.001435	0.515733
*PLAT*	C8S1799	0.005739	0.190653
*PLAT*	C8S1811	0.001435	0.0753783
*RARB*	C3S635	0.000717	0.653224
*RARB*	C3S679	0.005022	0.632142
*SIRT1*	C10S3048	0.002152	0.825893
*SIRT1*	C10S3050	0.002152	0.956865
*SIRT1*	C10S3058	0.000717	0.393157
*SIRT1*	C10S3092	0.000717	0.352589
*SIRT1*	C10S3093	0.000717	0.47264
*SIRT1*	C10S3107	0.000717	0.99946
*SIRT1*	C10S3108	0.000717	0.52925
*SIRT1*	C10S3109	0.000717	0.57047
*SIRT1*	C10S3110	0.002152	0.117719
*SREBF1*	C17S1007	0.002152	0.548739
*SREBF1*	C17S1009	0.000717	0.716057
*SREBF1*	C17S1024	0.004304	0.447239
*SREBF1*	C17S1030	0.000717	0.734055
*SREBF1*	C17S1043	0.004304	0.459494
*SREBF1*	C17S1045	0.003587	0.30998
*SREBF1*	C17S1046	0.002869	0.604567
*SREBF1*	C17S1048	0.001435	0.297328
*SREBF1*	C17S1055	0.001435	0.957889
*SREBF1*	C17S1056	0.000717	0.46384
*VLDLR*	C9S367	0.000717	0.510889
*VLDLR*	C9S376	0.002869	0.543897
*VLDLR*	C9S377	0.001435	1.20543
*VLDLR*	C9S391	0.000717	0.483147
*VLDLR*	C9S430	0.000717	0.677573
*VLDLR*	C9S443	0.001435	0.61953
*VLDLR*	C9S444	0.001435	0.901646
*VLDLR*	C9S497	0.000717	0.731422
*VNN1*	C6S5378	0.005739	0.466305
*VNN1*	C6S5380	0.170732	0.248606
*VNN3*	C6S5412	0.000717	0.551757
*VNN3*	C6S5426	0.032999	0.110779
*VNN3*	C6S5439	0.000717	0.127341
*VNN3*	C6S5441	0.098278	0.268411
*VNN3*	C6S5446	0.000717	0.528353
*VNN3*	C6S5448	0.000717	0.581462
*VNN3*	C6S5449	0.010043	0.680317
*VWF*	C12S181	0.000717	0.76848
*VWF*	C12S211	0.005739	0.337463

### Q4

Q4 had a heritability of 0.70, but none of this genetic component was due to genes in this sequencing set (i.e., it was not influenced by any of the genotyped exonic SNPs). Q4 was lower in smokers, decreased with age, and was lower in females. Q4 was protective; that is, individuals with higher levels of Q4 had lower risk of disease.

### Affection status

A normally distributed latent liability trait (not included in the distributed phenotype data) was simulated; it was influenced by 51 SNPs in 15 genes with 1–24 functional variants per gene (see Table 3). MAFs of these variants ranged from 0.07% to 25.8%. In all cases, the minor allele was associated with higher mean liability. This latent liability trait was also higher in smokers and increased with age. Disease risk was a function of this latent liability, Q1, Q2, and Q4:

**Table 3:** Effects on disease liability

Gene	SNP	MAF	*β*
*AKT3*	C1S11396	0.000717	0.340456
*BCL2L11*	C2S2286	0.000717	0.274592
*BCL2L11*	C2S2288	0.002869	0.563598
*BCL2L11*	C2S2307	0.000717	0.606816
*ELAVL4*	C1S3181	0.000717	0.64359
*ELAVL4*	C1S3182	0.000717	0.214219
*HSP90AA1*	C14S3630	0.000717	0.0579258
*HSP90AA1*	C14S3695	0.000717	0.152617
*HSP90AA1*	C14S3704	0.003587	0.0789197
*HSP90AA1*	C14S3706	0.258250	0.0874168
*NRAS*	C1S5748	0.000717	0.409806
*PIK3C2B*	C1S9164	0.001435	0.205094
*PIK3C2B*	C1S9165	0.000717	0.141183
*PIK3C2B*	C1S9172	0.004304	0.508901
*PIK3C2B*	C1S9173	0.001435	0.12026
*PIK3C2B*	C1S9174	0.000717	0.634406
*PIK3C2B*	C1S9189	0.006456	0.454308
*PIK3C2B*	C1S9200	0.000717	0.679158
*PIK3C2B*	C1S9222	0.000717	0.38177
*PIK3C2B*	C1S9250	0.001435	0.358232
*PIK3C2B*	C1S9266	0.002869	0.184476
*PIK3C2B*	C1S9267	0.002152	0.504508
*PIK3C2B*	C1S9306	0.000717	0.239692
*PIK3C2B*	C1S9320	0.000717	0.653693
*PIK3C2B*	C1S9333	0.000717	0.703217
*PIK3C2B*	C1S9346	0.000717	0.29823
*PIK3C2B*	C1S9373	0.000717	0.399922
*PIK3C2B*	C1S9391	0.000717	0.582382
*PIK3C2B*	C1S9423	0.000717	0.590111
*PIK3C2B*	C1S9432	0.010760	0.461306
*PIK3C2B*	C1S9445	0.000717	0.582247
*PIK3C2B*	C1S9446	0.000717	0.477664
*PIK3C2B*	C1S9449	0.000717	0.647146
*PIK3C2B*	C1S9455	0.002869	0.518095
*PIK3C2B*	C1S9457	0.000717	0.497112
*PIK3C3*	C18S2475	0.000717	0.695313
*PIK3C3*	C18S2492	0.017217	0.576351
*PIK3R3*	C1S2919	0.000717	0.414798
*PRKCA*	C17S4578	0.166428	0.39334
*PRKCA*	C17S4581	0.000717	0.129034
*PRKCB1*	C16S1894	0.000717	0.45754
*PTK2*	C8S4825	0.000717	0.0164796
*PTK2*	C8S4839	0.000717	0.142502
*PTK2B*	C8S886	0.000717	0.466067
*PTK2B*	C8S900	0.001435	0.111154
*PTK2B*	C8S909	0.001435	0.431062
*RRAS*	C19S4929	0.001435	0.34384
*RRAS*	C19S4937	0.001435	0.462103
*SHC1*	C1S7061	0.006456	0.206036
*SOS2*	C14S1381	0.000717	0.613801
*SOS2*	C14S1382	0.003587	0.633247

Liability to disease = latent liability + Q1 + Q2 – Q4. (1)

Using this formula, a quantitative liability score was calculated for each individual, and the top 30% of the distribution in each simulation replicate was declared affected. A consequence of this assignment was that each replicate had the same number of affected individuals, although the identity of these individuals varied across replicates. The effect sizes in Table 3 are for liability to disease.

## Competing interests

The authors declare that there are no competing interests.

## Authors’ contributions

LA, TDD, and JB designed the phenotypic trait model and the pedigree and unrelated individuals data sets. JMP aligned and processed sequence data. JCC and JEC provided input on incorporation of pathway and functional information and selection of functional variants. TDD conducted the gene dropping and phenotype simulations. JWK, TDD, LA, and JB performed error checking on the simulated data. All authors read and approved the final manuscript.

## References

[B1] The 1000 Genomes Project Consortium A map of human genome variation from population-scale sequencing Nature 2010 467 1061 1073 10.1038/nature09534 20981092 PMC3042601

[B2] McKenna A Hanna M Banks E Sivachenko A Cibulskis K Kernytsky A Garimella K Altshuler D Gabriel S Daly M The Genome Analysis Toolkit: a MapReduce framework for analyzing next-generation DNA sequencing data Genome Res 2010 20 1297 1303 10.1101/gr.107524.110 20644199 PMC2928508

[B3] Terwilliger JD Speer M Ott J Chromosome-based method for rapid computer simulation in human genetic linkage analysis Genet Epidemiol 1993 10 217 224 10.1002/gepi.1370100402 8224802

[B4] MacCluer JW Blangero J Dyer TD Speer MC GAW10: simulated family data for a common oligogenic disease with quantitative risk factors Genet Epidemiol 1997 14 737 742 10.1002/(SICI)1098-2272(1997)14:6<737::AID-GEPI29>3.0.CO;2-Q 9433570

[B5] Almasy L Terwilliger JD Nielsen D Dyer TD Zaykin D Blangero J GAW12: simulated genome scan, sequence, and family data for a common disease Genet Epidemiol 2001 21 suppl 2 S332 S338 11793693 10.1002/gepi.2001.21.s1.s332

